# Identification of key lncRNAs in colorectal cancer progression based on associated protein–protein interaction analysis

**DOI:** 10.1186/s12957-017-1211-7

**Published:** 2017-08-10

**Authors:** Haishan Zhu, Jiajing Yu, Haifeng Zhu, Yusheng Guo, Shengjie Feng

**Affiliations:** 1 The First Hospital of ZhaoQing, Guangdong, China; 2 Huashan Hospital, Fudan University, Shanghai, China

**Keywords:** Long non-coding RNA, Colorectal cancer, Protein–protein interaction analysis, Expression profiling

## Abstract

**Background:**

Colorectal cancer (CRC) was one of the most commonly diagnosed malignancies. The molecular mechanisms involved in the progression of CRC remain unclear. Accumulating evidences showed that long noncoding RNAs (lncRNAs) played key roles in tumorigenesis, cancer progression, and metastasis. Therefore, we aimed to explore the roles of lncRNAs in the progression of CRC.

**Methods:**

In this study, we aimed to identify differentially expressed lncRNAs and messenger RNAs (mRNAs) in CRC by analyzing a cohort of previously published datasets: GSE64857. GO and KEGG pathway analyses were applied to give us insight in the functions of those lncRNAs and mRNAs in CRC.

**Results:**

Totally, 46 lncRNAs were identified as differentially expressed between stage II and stage III CRC for the first time screening by microarray. GO and KEGG pathway analyses showed that differentially expressed lncRNAs were involved in regulating signal transduction, cell adhesion, cell differentiation, focal adhesion, and cell adhesion molecules.

**Conclusions:**

We found three lncRNAs (LOC100129973, PGM5-AS1, and TTTY10) widely co-expressed with differentially expressed mRNAs. We also constructed lncRNA-associated PPI in CRC and found that these lncRNAs may be associated with CRC progression. Moreover, we found that high PGM5-AS1 expression levels were associated with worse overall survival in CRC cancer. We believe that this study would provide novel potential therapeutic and prognostic targets for CRC.

**Electronic supplementary material:**

The online version of this article (doi:10.1186/s12957-017-1211-7) contains supplementary material, which is available to authorized users.

## Background

As the third most commonly diagnosed malignancy in most parts of the world, colorectal cancer (CRC) caused more than 600,000 deaths (approximately 8%) of all cancer deaths [1]. Due to lack of oncogenesis-associated molecular biomarkers, the overall survival time of CRC patients is still not improved remarkably after a mass of progress in clinical treatment for CRC [2]. Thus, to develop novel treatments of CRC, a more clear understanding of molecular mechanisms underlying the development and progression of CRC is urgently needed and new diagnostic and prognostic biomarkers are essential to be identified.

Endogenous cellular RNAs with lengths longer than 200 nucleotides and lack of obvious open reading frame (ORF) are the definition of long noncoding RNAs (also known as lncRNAs), which are lately discovered to be RNAs and make up 80% of noncoding RNAs [3, 4]. More than 8000 lncRNA genes are identified within 4 years, and the number of human lncRNAs are estimated ranging from 10,000 to 20,000 [5]. Although accumulating evidences showed that lncRNAs have been correlated to cancer progression including CRC, the functions of most lncRNAs are still unknown [3]. Concretely, lncRNA DANCR is a prognostic factor for both overall survival (OS) and disease-free survival (DFS) in CRC [6, 7]; upregulation of lncRNA FTX promoted growth, invasion, and migration in CRC cells [8, 9]; and the expression of lncRNA HOTAIR is associated with tumor invasion and radio-sensitivity suggested its potential role in CRC diagnostics and therapeutics [10, 11].

In this study, we aimed to identify differentially expressed lncRNAs and messenger RNAs (mRNAs) in CRC by analyzing a cohort of previously published datasets: GSE64857. To provide novel information about molecular mechanisms and functional roles of lncRNAs, we conducted protein–protein interaction analysis in CRC and found that several lncRNAs may be associated with the tumorigenesis of different CRC subtypes.

## Methods

### Microarray data and data preprocessing

Microarray data was downloaded from Gene Expression Omnibus (GEO) database (www.ncbi.nlm.nih.gov/geo/) under the accession number GSE64857. This dataset was acquired from the study by Wang [12]. Totally, 81 samples were included in this dataset, which consisted of 44 stage II and 37 stage III CRC samples. We used arrayQuality package to quality control and limma package to apply raw data in R software. The normalization criteria were quantile normalization. Genes having fold changes ≥2 and *P* values <0.05 were selected as of significantly differential expression.

### lncRNA classification pipeline

We applied a pipeline to evaluate the lncRNA expression in microarray data as previously described [13]. The following criteria were used to identify the uniquely probe sets for lncRNAs from the Affymetrix array. We retained Refseq IDs labeled as “NR_” (NR indicates non-coding RNA in the Refseq database). For the probe sets with Ensembl gene IDs, we retained those annotated with “lncRNA”, “processed transcripts”, “non-coding”, or “misc_RNA” in Ensembl annotations. Then, we filtered the probe sets obtained from the last step by filtering out pseudogenes, rRNAs, microRNAs, tRNAs, snRNAs, and snoRNAs. Finally, we got 2448 annotated lncRNA transcripts with corresponding Affymetrix probe IDs. lncRNAs having fold changes ≥2 and *P* values <0.05 were selected as of significantly differential expression.

### Co-expression network construction and analysis

In this study, the Pearson correlation coefficient of differentially expressed gene (DEG)-lncRNA pairs was calculated according to their expression value. We used the “cor” function in R software, which was a common software. All parameters are default values. The co-expressed DEG-lncRNA pairs with the absolute value of Pearson correlation coefficient ≥0.5 were selected, and the co-expression network was established by using cytoscape software.

### GO and KEGG pathway analyses

MAS system provided by CapitalBio company (Molecule Annotation System, http://bioinfo.capitalbio.com/mas3/) was used to determine the biological roles of differentially expressed mRNAs. Gene functions were classified into three subgroups namely BP (biological process), CC (cellular component), and MF (molecular function). The enriched GO terms were presented by enrichment scores. KEGG pathway analysis was carried out to determine the involvement of differentially expressed mRNAs in different biological pathways. The recommend *p* value (hypergeometric *P* value) cutoff is 0.05.

### Identification of lncRNA-associated PPI modules

STRING online software was used to analyze the interaction. The interaction relationships of the proteins encoded by DEGs were searched by STRING online software, and the combined score >0.4 was used as the cutoff criterion. The PPI network was visualized using Cytoscape software.

### Statistical analysis

The numerical data were presented as mean ± standard deviation (SD) of at least three determinations. Statistical comparisons between groups of normalized data were performed using *t* test or Mann–Whitney *U* test according to the test condition. The *p* < 0.05 was considered statistically significant with a 95% confidence level.

## Results

### Systematic analysis of the significantly differentially expressed mRNAs and lncRNA between stage II and stage III CRC

To identify the significantly differentially expressed mRNAs and lncRNA between stage II and stage III CRC, we utilized a publicly available gene expression data, GSE64857. We identified a total of 1472 DEGs (806 up- and 666 downregulated) and 46 differentially expressed lncRNAs (24 up- and 22 downregulated) in stage III CRC compared to stage II CRC samples (see Additional file 1). The top ten up- and downregulated lncRNAs were listed in Table 1.

**Table 1:** Top 10 up- and downregulated lncRNAs between stage II and stage III CRC identified by microarray analysis

ID	Gene symbol	R_p value	Fold change	Regulation
1557424_at	LOC100505878	0.003	0.457208687	Down
1562805_at	TLR8-AS1	0.000	0.51494527	Down
1562720_at	LOC101927286	0.002	0.521850269	Down
238180_at	LOC102724094	0.008	0.524416017	Down
1555822_at	FAM138A	0.001	0.540134279	Down
1564485_at	LINC00887	0.004	0.541149873	Down
1568854_at	LINC00240	0.029	0.542507891	Down
241394_at	LOC101928710	0.008	0.545171478	Down
1569330_at	STX18-AS1	0.004	0.557794767	Down
1557133_at	LINC00632	0.026	0.575279541	Down
236756_at	CENPVP1 /// CENPVP2	0.037	1.695335669	Up
1554666_at	LOC100130950	0.009	1.695717296	Up
215229_at	LOC100129973	0.033	1.702442591	Up
1569582_at	AADACP1	0.044	1.741719099	Up
221129_at	FAM215A	0.004	1.773586319	Up
230595_at	PGM5-AS1	0.041	1.780770219	Up
1561732_at	LOC101929181	0.006	1.827357207	Up
1562121_at	CHL1-AS1	0.010	1.862149071	Up
224293_at	TTTY10	0.000	2.254193266	Up
231898_x_at	SOX2-OT	0.002	2.483348784	Up

### Co-expression network analysis

**Fig. 1:**
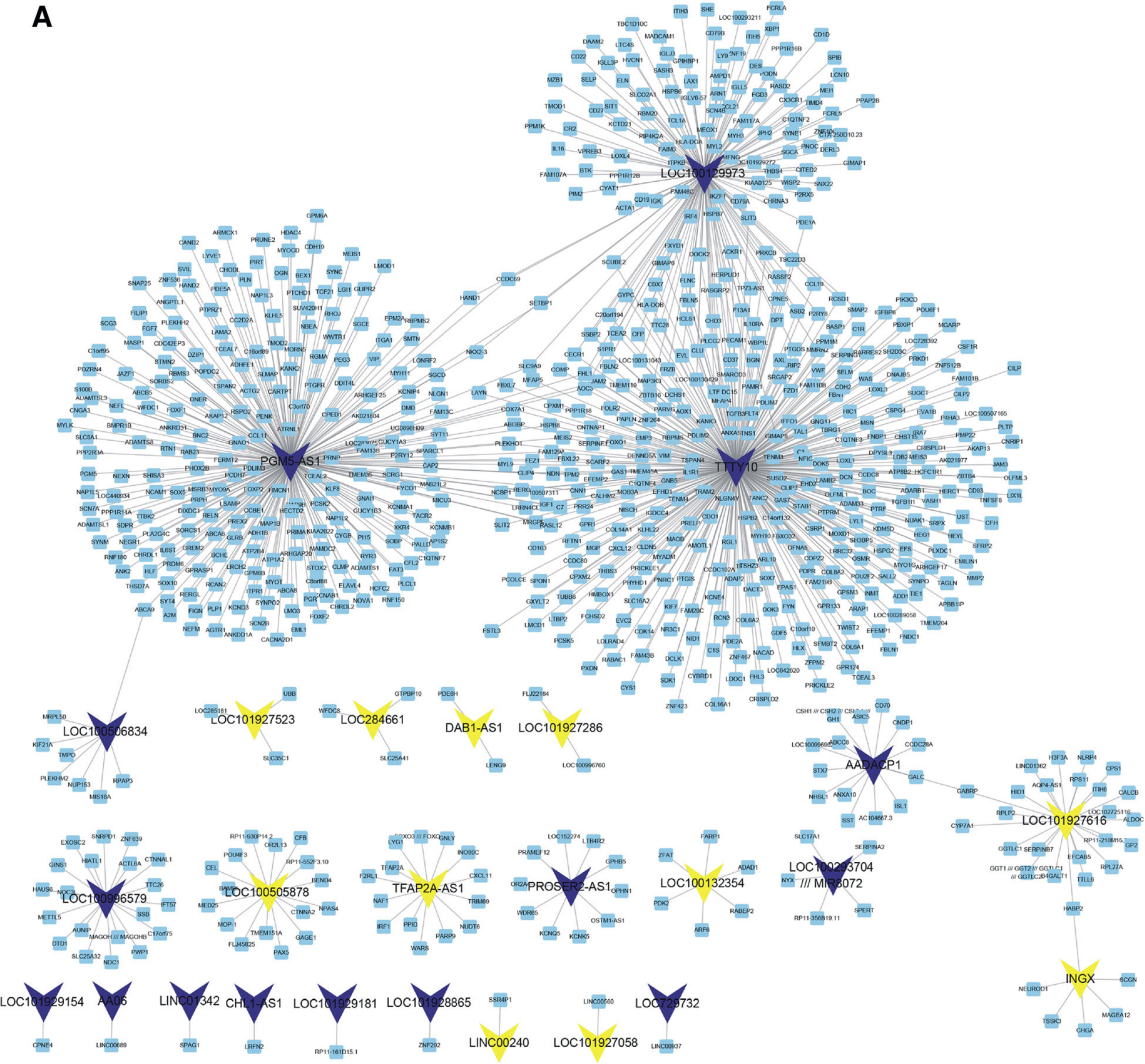
Construction of co-expressed DEG-lncRNA networks in colorectal cancer between stage II and stage III. **a** Totally, 46 lncRNAs and 881 differentially expressed genes were clustered in the network

To predict the potential functions of 24 up- and 22 downregulated lncRNAs, we first calculated the Pearson correlation coefficient of DEG-lncRNA pairs according to their expression value. The co-expressed DEG-lncRNA pairs with the absolute value of Pearson correlation coefficient ≥0.5 were selected. As shown in Fig. 1, the network included 46 lncRNAs and 881 differentially expressed genes (Fig. 1).

### GO and KEGG analyses of differentially expressed lncRNAs

**Fig. 2:**
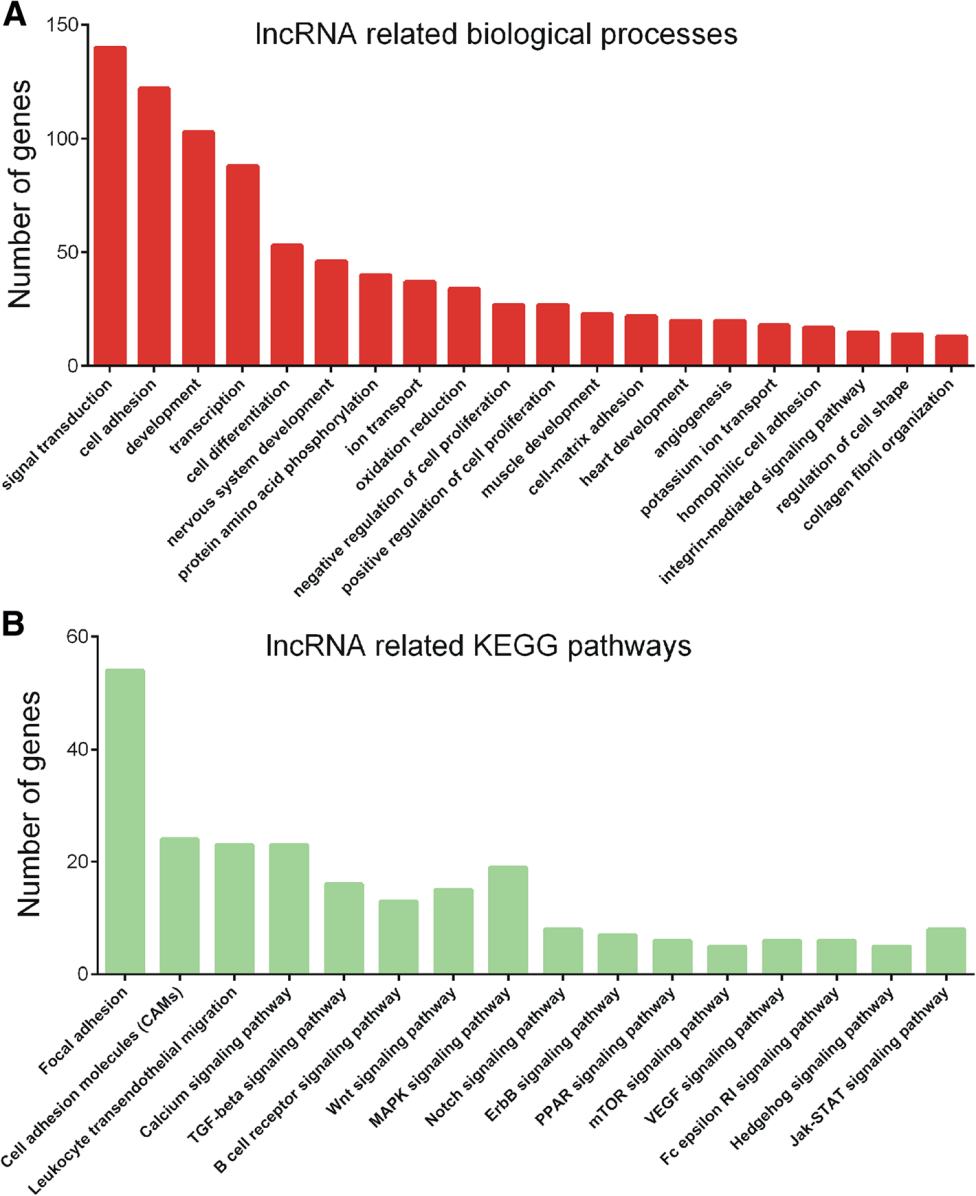
GO and KEGG analyses of differentially expressed lncRNAs in colorectal cancer between stage II and stage III. **a** GO pathway analysis of the dysregulated lncRNAs. **b** KEGG analysis of the dysregulated lncRNAs

Based on co-expression networks, we performed GO and KEGG analyses for differentially expressed lncRNAs by using the set of co-expressed mRNAs (Fig. 2a, b).

According to the GO analysis, differentially expressed lncRNAs were enriched in signal transduction, cell adhesion, development, transcription, cell differentiation, and cell proliferation. KEGG pathway analysis revealed that differentially expressed lncRNAs mainly participated in regulating focal adhesion, cell adhesion molecules, calcium signaling pathway, and TGF-beta signaling pathway.

### lncRNA co-expressed mRNAs was connected by PPI

In this study, we found three upregulated lncRNAs (LOC100129973, PGM5-AS1, and TTTY10) could widely co-expressed with DEGs. Among them, PGM5-AS1 co-expressed with more than 275 DEGs, LOC100129973 co-expressed with about 200 DEGs, and TTTY10 co-expressed with about 350 DEGs in the GSE64857 data. Next, we analyzed the co-expressed mRNAs of these lncRNAs and examined whether the mRNAs were connected by PPIs.

**Fig. 3:**
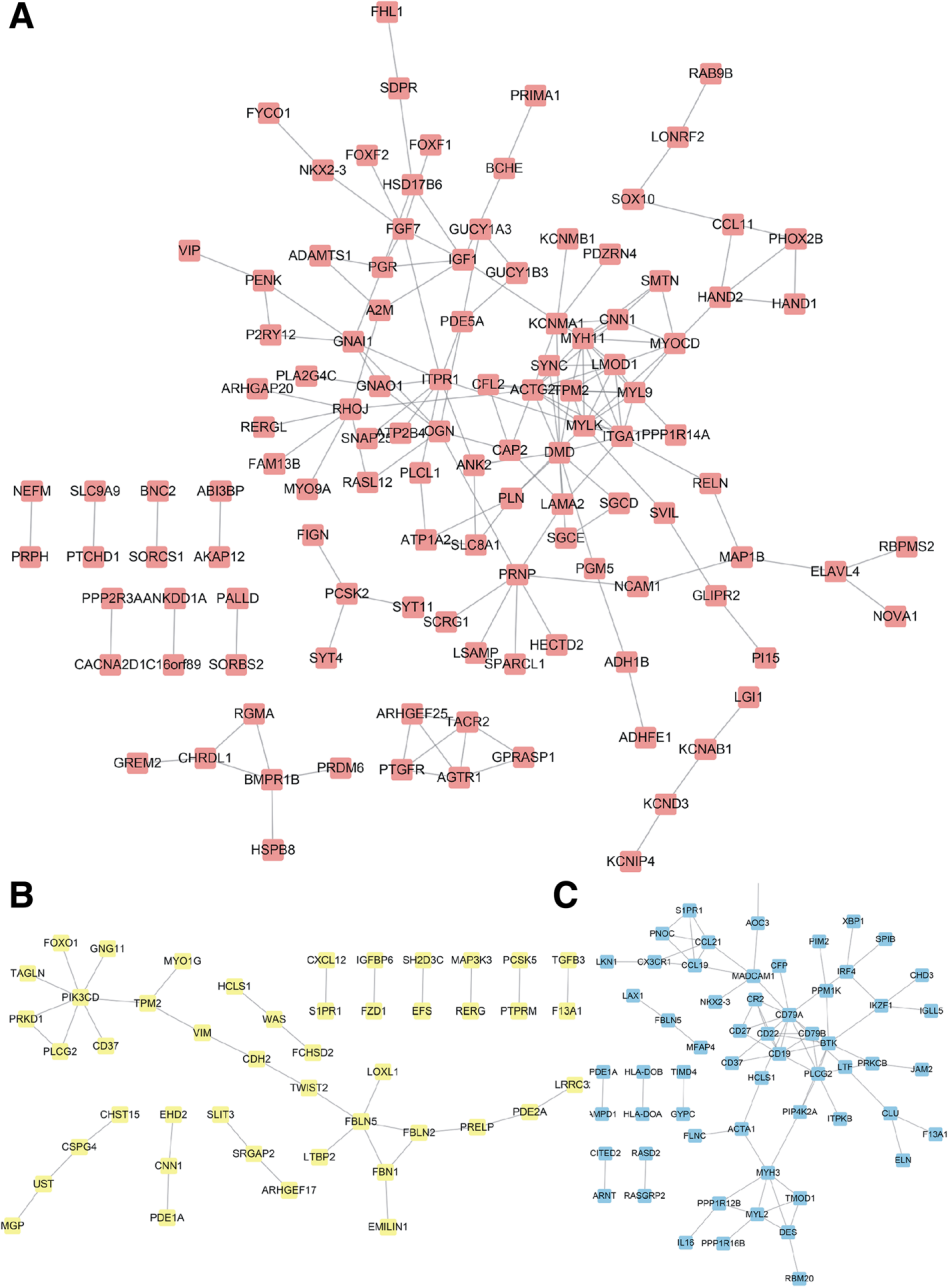
Construction of PPI network of lncRNA (PGM5-AS2, TTTY10, and LOC100129973) co-expressed mRNAs in colorectal cancer between stage II and stage III. **a** The PGM5-AS1-related PPI network in colorectal cancer. **b** The TTTY10-related PPI network in colorectal cancer. **c** The LOC100129973-related PPI network in colorectal cancer

Based on the information in the STRING database, we constructed a protein–protein interaction network of each lncRNA in the CRC. The PGM5-AS1-related PPI network contained 72 nodes and 163 edges, and the hub nodes with the highest connectivity degree were ACTG2 (degree = 12), DMD (degree = 11), MYLK (degree = 11), and MYH11 (degree = 10) (Fig. 3a). The TTTY10-related PPI network contained 26 nodes and 37 edges, and the hub nodes with the highest connectivity degree were PIK3CD (degree = 7) (Fig. 3b). The LOC100129973-related PPI network contained 41 nodes and 78 edges, and the hub nodes with the highest connectivity degree were CD79A (degree = 11) (Fig. 3c).

### Exploring the molecular functions of PGM5-AS1, TTTY10, and LOC100129973

**Fig. 4:**
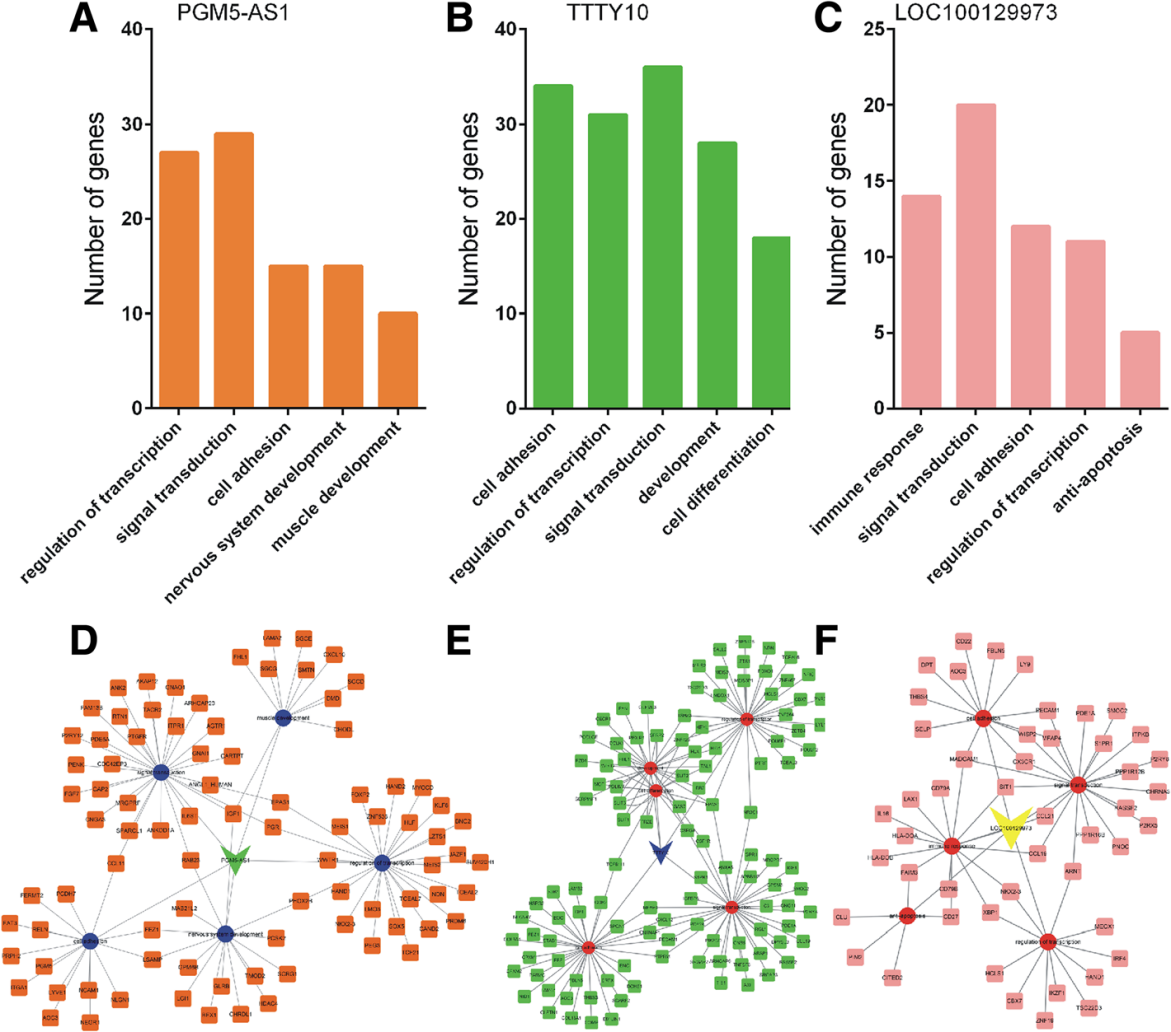
GO analysis of lncRNAs PGM5-AS1, TTTY10, and LOC100129973 in colorectal cancer between stage II and stage III. GO pathway analysis of lncRNAs PGM5-AS1 (**a**, **d**), TTTY10 (**b**, **e**) and (**c**, **f**) LOC100129973 (**b**, **e**)

The molecular functions of LOC100129973, PGM5-AS1, and TTTY10 in the CRC progression were still unknown. To further explore the molecular function of LOC100129973, PGM5-AS1, and TTTY10, we perform GO analysis of them using their co-expressed genes. We found that PGM5-AS1 was associated with the regulation of transcription, signal transduction, cell adhesion, nervous system development, and muscle development (Fig. 4a, d). TTTY10 was associated with cell adhesion, regulation of transcription, signal transduction, development, and cell differentiation (Fig. 4b, e). LOC100129973 was associated with immune response, signal transduction, cell adhesion, regulation of transcription, and anti-apoptosis (Fig. 4c, f).

### Alterations of PGM5-AS1 expression and prognosis in CRC

**Fig. 5:**
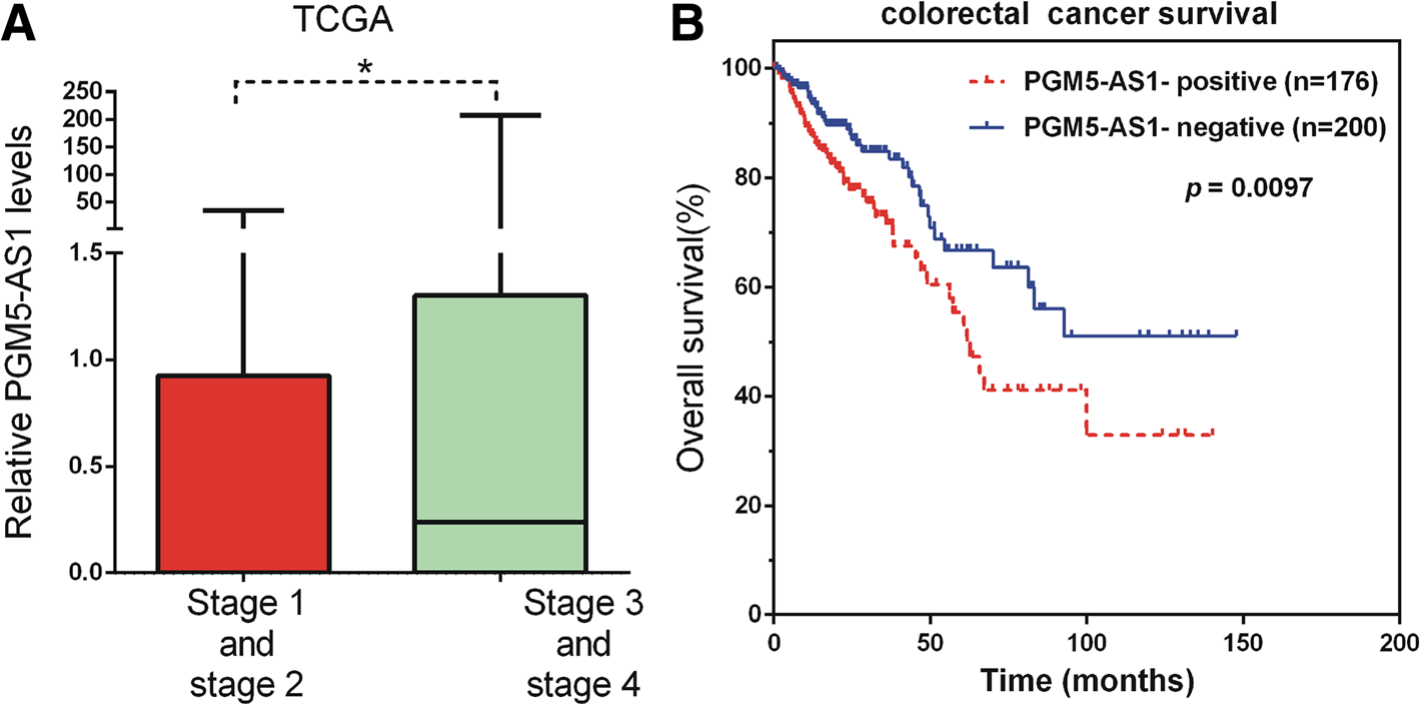
PGM5-AS1 in TCGA database. **a** The expression levels of PGM5-AS1 in stage III and IV CRC samples. **b** The overall survival rate of high PGM5-AS1 level and low PGM5-AS1 level patients

To evaluate possible prognostic value of PGM5-AS1, TTTY10, and LOC100129973, we download the RNA-seq data from cbioportal (http://www.cbioportal.org/). However, only PGM5-AS1 (LOC100129973 was not included in TCGA, and TTTY10 expression was too low) expression levels with survival data were available to analyze. As shown in Fig. 5a, we also observed that PGM5-AS1 were upregulated in stage III and IV CRC samples. Kaplan–Meier analysis showed patients with high PGM5-AS1 expression levels had decreased overall survival compared to those with low PGM5-AS1 levels (*p* = 0.0097).

## Discussion

The molecular mechanism involved in the CRC progression remained unclear. Therefore, it was critically important to investigate the biological mechanisms of CRC. In the present study, we identified the significantly differentially expressed mRNAs and lncRNAs between stage II and stage III CRC by using GSE64857. GO and KEGG pathway analyses showed that differentially expressed lncRNAs were involved in regulating CRC progression. Our analysis also revealed that the function of co-expressed mRNAs related to PGM5-AS1, TTTY10, and LOC100129973 could be connected by PPI.

CRC was one of the deadliest malignancies due to its lack of biomarkers for early diagnosis and efficient therapeutic strategies [14]. Recently, studies had shown that lncRNAs played key roles in tumorigenesis, cancer progression, and metastasis. Increasingly, reports also demonstrated that lncRNAs’ expression could be deregulated in human cancers, including CRC [15–17]. In the present study, we identified the significantly differentially expressed mRNAs and lncRNAs between stage II and stage III CRC using a publicly available gene expression data, GSE64857. From the microarray expression profiles, we identified 1472 DEGs (806 up- and 666 downregulated) and 46 differentially expressed lncRNAs (24 up- and 22 downregulated) in stage III CRC compared to stage II CRC samples altogether.

One challenge in predicting the functions of lncRNAs is that lncRNA could not be catalogued by GO and KEGG pathways analyses directly. According to the report of Guttman et al., one approach to classify the putative function of ncRNAs uses “guilt-by-association” [18]. In the previous reports, combination co-expression with GO analysis were widely used to predict lncRNAs’ functions in triple-negative breast cancer [19] and prostate cancer [20]. To predict the functions of the differentially expressed lncRNAs, we first constructed co-expression networks and performed GO and KEGG analyses for differentially expressed lncRNAs according to Guttman’s report. According to the GO analysis, differentially expressed lncRNAs were enriched in signal transduction, cell adhesion, development, transcription, cell differentiation, and cell proliferation. KEGG pathway analysis revealed that differentially expressed lncRNAs mainly participated in regulating focal adhesion, cell adhesion molecules, calcium signaling pathway, and TGF-beta signaling pathway.

Recently, several reports had shown that altered expression of lncRNAs may have important mechanisms of CRC progression. A few lncRNAs including DANCR [11], FTX [9, 15], and HOTAIR were significantly associated with the progression of CRC [21, 22]. However, the molecular mechanisms and functional roles underlying the lncRNAs in transformation of CRC remain largely unknown. In this study, we identified three upregulated lncRNAs (LOC100129973, PGM5-AS1, and TTTY10) could widely co-express with DEGs. lncRNA LOC100129973 was reported to suppress apoptosis in vascular endothelial cells by targeting miR-4707-5p and miR-4767 [23]. However, the molecular function of LOC100129973, PGM5-AS1, and TTTY10C remains unclear in CRC. Here, to explore their molecular mechanisms, we analyzed the co-expressed mRNAs of these lncRNAs and examined whether the mRNAs were connected by PPIs. We found that PGM5-AS1 was associated with the regulation of transcription, signal transduction, and cell adhesion. Interestingly, we found PGM5-AS1 may regulate cell adhesion by effecting PGM5. PGM5 was a kind of phosphotransferase involved in the interconversion of glucose-1-phosphate and glucose-6-phosphate. In CRC, PGM5 was also reported as a potential protein marker of colorectal adenoma [24]. TTTY10 was identified to be involved in regulating cell adhesion, transcription, signal transduction, development, and cell differentiation by regulating FOXO1 [25], SLIT1, and SLIT3 [26]. We observed that LOC100129973 was associated with immune response, signal transduction, cell adhesion, regulation of transcription, and anti-apoptosis and suggested that LOC100129973 was involved in regulating CRC proliferation. To evaluate possible prognostic value of PGM5-AS1, TTTY10, and LOC100129973, we analyzed the TCGA data and found high PGM5-AS1 expression levels were associated with worse overall survival in CRC cancer.

## Conclusion

In conclusion, we identified differentially expressed lncRNAs between stage II and stage III CRC for the first time screened by microarray. We found that 46 lncRNAs were dysregulated in CRC totally. GO and KEGG pathway analyses showed that differentially expressed lncRNAs were involved in regulating signal transduction, cell adhesion, cell differentiation, focal adhesion, and cell adhesion molecules. Three lncRNAs (LOC100129973, PGM5-AS1, and TTTY10) were identified to widely co-express with DEGs. We also constructed lncRNA-associated PPI in CRC. Of note, we observed that high PGM5-AS1 expression levels were associated with worse overall survival in CRC cancer. We believed that this study would provide novel potential therapeutic and prognostic targets for CRC.

## Additional file

Additional file 1: Table S1. Differentially expressed lncRNAs between stage II and stage III CRC identified by microarray analysis. (XLSX 12 kb)

## Abbreviations

CRC: Colorectal cancer
DEG: Differentially expressed gene
DFS: Disease-free survival
GEO: Gene expression omnibus
ORF: Open reading frame
OS: Overall survival
SD: Standard deviation
